# Emergence of quinolone resistance among extended-spectrum beta-lactamase-producing *Enterobacteriaceae *in the Central African Republic: genetic characterization

**DOI:** 10.1186/1756-0500-4-309

**Published:** 2011-08-25

**Authors:** Thierry Frank, Jean R Mbecko, Pembe Misatou, Didier Monchy

**Affiliations:** 1 Service de Bactériologie, Institut Pasteur de Bangui, Bangui, Central African Republic

**Keywords:** Quinolones, Betalactams, Antibiotic resistance, *Enterobacteriaceae*

## Abstract

**Background:**

Cross-resistance to quinolones and beta-lactams is frequent in *Enterobacteriaceae*, due to the wide use of these antibiotics clinically and in the food industry. Prescription of one of these categories of antibiotic may consequently select for bacteria resistant to both categories. Genetic mechanisms of resistance may be secondary to a chromosomal mutation located in quinolone resistance determining region of DNA gyrase or topoisomerase IV or to a plasmid acquisition. The insertion sequence IS*CR1 *is often associated with *qnr *and may favour its dissemination in Gram-negative bacteria. The aim of this study was to determine the genetic mechanism of quinolone resistance among extended-spectrum beta-lactamase-producing *Enterobacteriaceae *strains in the Central African Republic.

**Findings:**

Among seventeen ESBL-producing *Enterobacteriaceae *isolated from urine, pus or stool between January 2003 and October 2005 in the Central African Republic, nine were resistant to ciprofloxacin (seven from community patients and two from hospitalized patients). The ESBL were previously characterized as CTX-M-15 and SHV-12. Susceptibility to nalidixic acid, norfloxacin and ciprofloxacin, and the minimal inhibitory concentrations of these drugs were determined by disc diffusion and agar dilution methods, respectively. The presence of plasmid-borne IS*CR1-qnrA *region was determined by PCR and amplicons, if any, were sent for sequencing. Quinolone resistance determining region of DNA gyrase *gyrA *gene was amplified by PCR and then sequenced for mutation characterization. We found that all CTX-M-producing strains were resistant to the tested quinolones. All the isolates had the same nucleotide mutation at codon 83 of *gyrA*. Two *Escherichia coli *strains with the highest MICs were shown to harbour an IS*CR1-qnrA1 *sequence. This genetic association might favour dissemination of resistance to quinolone and perhaps other antibiotics among *Enterobacteriaceae*.

**Conclusions:**

This study shows that at least two mechanisms might explain the emerging resistance of *Enterobacteriaceae *to quinolones in the CAR. Beside the classical topoisomerase mutation, the cause may be acquisition of a plasmid-borne *qnrA1*. Clinicians and bacteriologists should be made aware of possible dissemination of IS*CR1-qnrA1 *among *Enterobacteriacae*.

## Background

Quinolones and beta-lactams are widely used antibiotics for the treatment of bacterial infections and are also extensively used in the food-processing industry. Their wide use has triggered increased bacterial resistance worldwide [1]. Quinolone resistance and extended-spectrum beta-lactamase (ESBL) production are often associated in *Enterobacteriaceae *[1]. The prescription of quinolones may consequently select bacteria that are not only quinolone-resistant but also beta-lactam-resistant and vice versa. The biological mechanisms of resistance to quinolones include impermeability, active efflux, target modification and antibiotic neutralization. Genetic determination of quinolone resistance is generally linked to chromosomal mutations affecting the quinolone resistance-determining regions (QRDR) of DNA gyrase (GyrA and GyrB) and topoisomerase IV (ParC and ParE) [1]. Mutations in *gyrA*, the gene encoding for the A subunit of DNA gyrase are the most common mechanism involved in quinolone resistance among Gram negative bacteria [2].

In 1994, the plasmid-borne quinolone resistance genes *qnr *were identified [1]. The insertion sequence IS*CR1 *has often been characterized in the genetic environment of *qnr *and may be involved in dissemination of the gene in Gram-negative bacteria [3].

Quinolone resistance was first identified in the CAR in 2003, among clinical ESBL-producing *Enterobacteriaceae*. The aim of this study was to determine the genetic mechanism of quinolone resistance of these strains isolated in the Central African Republic (CAR).

## Methods

Of the seventeen ESBL-producing *Enterobacteriaceae *isolated from urine, pus or stool samples between January 2003 and October 2005 at the Institut Pasteur de Bangui and previously characterized as CTX-M-15 and SHV-12, nine were resistant to ciprofloxacin (eight *Escherichia coli *and one *Klebsiella pneumoniae*) [4]. Seven *E.coli *strains were isolated from community patients and the two others were isolated from hospitalized patients.

Antibiotic susceptibility was determined by the diffusion disc method (Comité de l'Antibiogramme de la Société Française de Microbiologie). The minimal inhibitory concentrations (MICs) of nalidixic acid, norfloxacin and ciprofloxacin were measured by the agar dilution method.

PCR was carried out on genomic DNA extracted with the commercial Qiagen DNA Mini Kit (Qiagen, Courtaboeuf, France) at 94 °C for 5 min, 35 cycles at 94 °C for 30 s, 55 °C for 40 s and extension at 72 °C for 60 s, followed by 72 °C for 7 min.

The ciprofloxacin-resistant isolates were screened for the *qnrA *gene by PCR with specific primers targeting IS*CR1*, 5'-TGAAACAGAAAACAGCCAAGG-3' (IS*CR1*qnr upper), and *qnrA*, 5'-GCAGCACTATTACTCCAAGG-3' (IS*CR1*qnr lower). The primers targeting IS*CR1-qnrA1 *were designed with Oligo-4 software. The sequences encoding QRDR of DNA gyrase were amplified with *gyrA6 *5'-CGA CCT TGC GAG AGA AAT-3' and *gyrA631R *5'-GTT CCA TCA GCC CTT CAA-3' [5].

All amplicons were sequenced at MWG Biotech France (http://www.mwg-biotech.com) and analysed with program ChromasPro 2.0 and blastN software (http://gb-admin@ncbi.nlm.nih.gov) (GenBank accession numbers for the sequenced IS*CR1-qnrA1 *region are JF728153 and JF728154).

All patients have given their agreement to participate in this study prior the samples collection. This study was reviewed and approved by the scientific committee board of University of Bangui in charge of the validation of scientific protocol studies in the CAR.

## Results and discussion

Susceptibility testing showed that all CTX-M-producing strains were resistant to nalidixic acid, norfloxacin and ciprofloxacin. The MIC of nalidixic acid and norfloxacin was 64 μg/ml and that of ciprofloxacin was 32 μg/ml for six *E. coli *and the *K. pneumoniae *strains. For two *E. coli *isolates, the MICs were higher, and PCR targeting IS*CR1-qnrA *amplified a 1050-bp fragment from these two isolates which was shown by DNA sequence analysis to be IS*CR1-qnrA1*. We could not determine the MICs values of the two *qnrA*-positive *E. coli *by the agar dilution method because they exceeded the highest concentrations of antibiotics tested in this study (32 μg/ml for ciprofloxacin and 64 μg/ml for the nalidixic acid). Other publications have shown that *qnrA *increased the MICs of quinolones by 10-fold in *Enterobacteriaceae *[6]. The combination of an insertion sequence IS*CR1 *with *qnrA1 *could ensure dissemination of quinolone resistance to both ESBL-producing and ESBL-negative *Enterobacteriaceae*. The IS*CR1 *element is also capable of mobilizing *qnrA1 *in multiple drug resistance regions, generally located on plasmids that mediate resistance to multiple antibiotics.

Sequence analysis of the gene portion encoding the QRDR of GyrA showed the same amino acid change in GyrA (Ser83-Leu) in all our isolates. Nucleotide mutations at codon 83 are the commonest GyrA substitutions found in quinolone-resistant *E. coli *and the serine residue is most commonly substituted by a leucine [7].

## Conclusion

We have shown that the emerging resistance of *Enterobacteriaceae *to quinolones in the CAR may be due to a topoisomerase mutation or a plasmid-borne *qnrA1 *acquisition. It would be interesting to sequence the DNA gyrase *gyrB *and topoisomerase IV *parC *and *parE *genes, to screen for the other *qnr *genes (*B *and *S*) and to characterize the environment of IS*CR1-qnrA1*, which has already been found in several *sul1-*type complex integrons. The possible dissemination of IS*CR1-qnrA1 *among *Enterobacteriacae *requires vigilance by bacteriology laboratories in the CAR. It will also be important to inform clinicians of the potential risk associated with prescribing quinolones and beta-lactams.

## Competing interests

The authors declare that they have no competing interests.

## Authors' contributions

TF: carried out the molecular genetic studies, validated the results of antibiotic susceptibility.testing, participated in nucleotide sequence analysis and drafted the manuscript.

JRM: participated in bacteriological isolation of *Enterobacteriaceae *strains. PM: participated in bacteriological isolation of *Enterobacteriaceae *strains. DM: helped to draft the manuscript. All authors read and approved the final manuscript.
